# Genome-scale reconstruction of *Paenarthrobacter aurescens* TC1 metabolic model towards the study of atrazine bioremediation

**DOI:** 10.1038/s41598-020-69509-7

**Published:** 2020-08-03

**Authors:** Shany Ofaim, Raphy Zarecki, Seema Porob, Daniella Gat, Tamar Lahav, Yechezkel Kashi, Radi Aly, Hanan Eizenberg, Zeev Ronen, Shiri Freilich

**Affiliations:** 1 Newe Ya’ar Research Center, Agricultural Research Organization, Ramat Yishay, Israel; 2 Faculty of Biotechnology and Food Engineering, Technion-Israel Institute of Technology, Haifa, Israel; 3 Department of Environmental Hydrology and Microbiology, Zuckerberg Institute for Water Research, Jacob Blaustein Institutes for Desert Research, Ben-Gurion University of the Negev, Midreshet Ben-Gurion, 8499000 Beersheba, Israel

**Keywords:** Computational biology and bioinformatics, Biochemical reaction networks, Computational models, Ecology, Microbiology, Environmental microbiology

## Abstract

Atrazine is an herbicide and a pollutant of great environmental concern that is naturally biodegraded by microbial communities. *Paenarthrobacter aurescens* TC1 is one of the most studied degraders of this herbicide. Here, we developed a genome scale metabolic model for *P. aurescens* TC1, *i*RZ1179, to study the atrazine degradation process at organism level. Constraint based flux balance analysis and time dependent simulations were used to explore the organism’s phenotypic landscape. Simulations aimed at designing media optimized for supporting growth and enhancing degradation, by passing the need in strain design via genetic modifications. Growth and degradation simulations were carried with more than 100 compounds consumed by *P. aurescens* TC1. In vitro validation confirmed the predicted classification of different compounds as efficient, moderate or poor stimulators of growth. Simulations successfully captured previous reports on the use of glucose and phosphate as bio-stimulators of atrazine degradation, supported by in vitro validation. Model predictions can go beyond supplementing the medium with a single compound and can predict the growth outcomes for higher complexity combinations. Hence, the analysis demonstrates that the exhaustive power of the genome scale metabolic reconstruction allows capturing complexities that are beyond common biochemical expertise and knowledge and further support the importance of computational platforms for the educated design of complex media. The model presented here can potentially serve as a predictive tool towards achieving optimal biodegradation efficiencies and for the development of ecologically friendly solutions for pollutant degradation.

## Introduction

Atrazine (2-chloro-4-ethylamino-6-isopropylamino-1,3,5-triazine) is an herbicide employed to control broadleaf and grass mainly in crops such as rice, wheat, maize, and sorghum. It is also a well-known pollutant of great environmental concern. Atrazine has been shown to have negative effects such as DNA damage, gene expression shifts, cancer and endocrine disruption^1,2^. Its residues are found in soil samples decades after it was last applied and were shown to chronically leach into local aquifers^3^. As such, efforts are being made to limit and monitor its use^4^. While atrazine was banned in the European Union and Switzerland since 2003, the United States Environmental Protection Agency still allows its wide use under monitoring^5,6^. Areas contaminated with atrazine and other hazardous herbicides are rapidly increasing worldwide introducing a need in remediation approaches. Bioremediation—an environmental bioprocess in which naturally occurring organisms are used for breaking down hazardous substances into less toxic or non-toxic substances—is increasingly acknowledged as a cost-effective feasible alternative for environmental cleaning^7–9^. Critical environmental bioprocesses are naturally carried out by bacteria and are related to removal of pollutants from water, soil or air^10^. Thus, bacterial bioremediation is widely applied for the degradation of various organic pollutants^11–14^ including herbicides^14–18^. Bioremediation may occur naturally or through the stimulating addition of fertilizers, electron acceptors, etc. These amendments encourage the growth of the degrading microbes within the medium or environment in a process termed biostimulation^8,19^. Microbial metabolism has been described as most influential on atrazine degradation, promoting the development of biodegradation strategies^20^. One of the well-known atrazine degraders is the gram-positive *Paenarthrobacter aurescens* TC1^21–25^ (previously known as *Arthrobacter aurescens* TC1), reported to biodegrade atrazine more efficiently than most other known degraders including *Pseudomonas* sp. ADP^26^. The full hydrolytic pathway for atrazine degradation in *P. aurescens* TC1 was described and reported^27^. While *P.* ADP can only use the hydrolytic products of the ring cleavage as a source of nitrogen but not as a source of carbon; TC1 can use the side groups of atrazine as source of carbon, nitrogen and energy consuming up to 3,000 mg of atrazine per liter^24^. As such, *P. aurescens* TC1 is an ideal candidate for the study of atrazine bioremediation using a genome scale metabolic model reconstruction.

Modern tools of genomics, transcriptomics, metabolomics, proteomics, signaling systems and synthetic biology have opened new avenues for biotechnological advances and are increasingly applied for promoting better understanding of complex biological systems. In recent years, constraint based metabolic modelling approaches have become widely used as an in silico tool for organism-level phenotyping and the subsequent development of metabolic engineering strategies^28,29^. Generally, such approaches follow four key steps: (1) data acquisition—mainly genome sequencing information, basic cell-physiological and biochemical knowledge and some experimental data on cell growth; (2) model reconstruction—translating data into structured mathematical representation; (3) constraint-based optimization simulations—the prediction of growth rate, substrate uptake rates, and byproduct rates under different growth conditions or following knockout mutations, in the absence of kinetic information^30–36^; and (4) experimental validation. The potential of such models for the investigation of optimal processing is now acknowledged and practiced^10,37–40^. Examples for applicative use include the optimal production or utilization of industrial compounds such as xylose^41^, biofuels^42^, vitamins^43^ and drug development^44^. In bioremediation, genome scale metabolic modeling approaches were applied for the design of *Geobacter sulfurreducens* strain capable of increased electron transfer and a higher Fe(III) respiratory that was beneficial for environmental bioremediation of uranium-contaminated groundwater^45^. Application of genome scale metabolic modeling for strain design is often based on the Optknock algorithm^46^ and its derivatives which search for the optimal gene knockouts for a desired metabolic production. However, unlike industrial use of engineered strain for enhanced production in monoculture, introduction of exogenous species into natural habitat is far from trivial and hampers the application of modeling approaches towards bioremediation solutions. An alternative strategy is the use of modeling for the design of optimal conditions that will enhance processes (degradation or synthesis) carried by the endogenous community^47^. For example, interactions between *Geobacter* and *Rhodoferax*—a competitor Fe(III)-reducers in anoxic subsurface environments—were captured and described using metabolic modelling and allowed identification of optimal degradation conditions^48^. Models can also predict the benefits of cooperative and commensal interactions to degradation efficiency. Xu et al. have demonstrated the use of metabolic models for predicting interactions between *P. aurescens* TC1 and endogenous soil species^49^. Exchange metabolites secreted by non-degraders species contribute for enhancing *P. aurescens* TC1 growth and expediting degradation efficiency^49,50^. Here, we directly focused on modeling-based identification of media supplements that can enhance atrazine degradation. We hypothesize that the introduction of specific nutrients into the bacteria’s immediate environment can resemble metabolic exchanges between *P. aurescens* TC1 and non-degrader species in soil and can lead to an increase in the total biomass and a subsequent increase in the degradation activity. We describe the process of reconstruction of a genome-scale metabolic model of *P. aurescens* TC1 and its evaluation as a predictive tool for the fast and low-cost screening of potential nutritional supplements that can serve as bio-stimulators of degradation.

## Results

### *Paenarthrobacter aurescens* TC1 genome scale metabolic network reconstruction

The genome of *P. aurescens* TC1 is composed of a circular chromosome of 4.6 Mb coding for 4,222 open reading frames (ORFs) as well as two plasmids, 0.3 Mb each, coding for additional ~ 600 ORFs^51^. The genome and plasmids were annotated and used to create a draft model reconstruction using the Model SEED pipeline^52^. The draft reconstruction included an automatic gap-filling step, using default (optimal) media options^52^. This initial reconstruction contained a list of gene-protein*-*reaction associations that were classified as exchange, transport, and cytosolic as well as a list of all relevant metabolites and a biomass reaction. Based on taxonomy, the biomass reaction was defined to describe a gram-positive bacteria^52^. The composition of the biomass reaction summarizes the fractional contribution of generalized microbial biomass precursors (e.g., amino acids and lipids) to the synthesis of a new cell and is similar to the previously published genome scale reconstruction of *Bacillus subtilis*^53^. Unspecified non-metabolic consuming processes in the cell were defined as ATP maintenance and set similarly to Oh et al.^53^.

**Figure 1:**
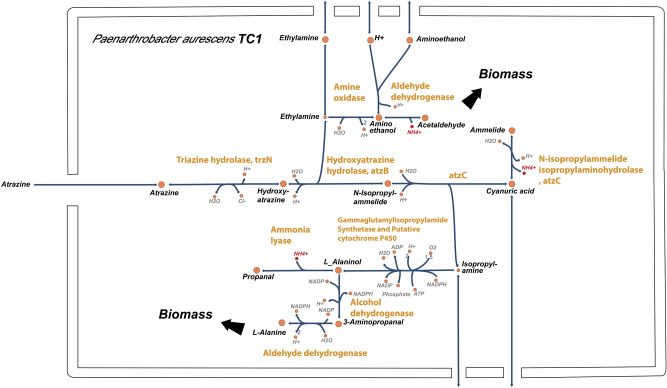
Atrazine metabolism as implemented in the cytoplasm compartment of *P. aurescens* TC1 genome scale metabolic model reconstruction (*i*RZ1179). Reaction (enzyme) names are written in orange.

Following automatic construction, several key steps of model refinement^54^ were carried out. The model's metabolic functions were compared to genome annotations of *P. aurescens* TC1 from four established and comprehensive public resources and databases (RAST^55^, KEGG^56^, JGI^57^ and UniProt^58^) and missing reactions were added to the draft network. All reactions were screened to verify that they are stoichiometrically balanced. Errors in reaction elemental balance and directionalities were determined according to the KEGG scheme. Model Reactions involved in atrazine degradation pathway and its further conversion into the cellular building blocks were added manually (Fig. 1) based on the detailed reports of the relevant pathways in the specific strain^24,59^. *P. aurescens* TC1 degrades atrazine by hydrolytic dechlorination. The process is catalyzed by triazine hydrolase (TrzN) followed by two hydrolytic deamination reactions catalyzed by hydroxyatrazine hydrolase (AtzB) and N-isopropylammelide isopropylamino hydrolase (AtzC). These enzymes convert atrazine to cyanuric acid and release the alkylamines ethylamine and isopropylamine. Cyanuric acid accumulates stoichiometrically, whereas the alkylamines are further catabolized into cellular building blocks^27^. Ethylamine and isopropylamine are converted into acetaldehyde and l-alanine (Fig. 1), respectively^59^, and can serve as sole sources of carbon and nitrogen required for growth^24^.

Initial simulations were carried for debugging and removing futile or erroneously energy generating loops. To this end, all external fluxes were blocked (upper and lower bounds set to zero). Next, a minimal medium was used, verifying that growth requires the supply of both carbon and nitrogen sources. After establishing no growth under infeasible conditions, we tested growth (biomass production) under experimentally verified conditions as described in Strong et al.^24^. Fine tuning of growth simulations to correctly represent the bacteria’s biology was done by manual curation. This included manual gap filling (addition of spontaneous and literature supported reactions) and curation of reaction directionality.

The reconstructed metabolic network presented here covers 25% of the ORFs present in the genome and includes 1,179 genes, 2,541 reactions, out of which 134 are exchange, and 2,848 metabolites. The coverage of the genome is similar to those of previously published reconstructed metabolic networks of gram-positive bacteria such as *C. difficile* or *B. subtilis* (20%)^53,60^ and the overall number of reactions is similar to the number of reactions covered by gold standard, updated models of species with similar genome sizes such as *i*ML1515^61^. A detailed description of the network including the reactions, metabolites, genes, and compartments that comprise the network is provided in Supplemental Dataset 1. The model is also available as Systems Biology Markup Language (SBML) file^62^ in Supplemental Dataset 2. The SBML file can be used with tools such as MATLAB or other SBML compliant software. The minimal media used for simulations are available in Supplemental Dataset 3.

### *i*RZ1179 validation

**Figure 2:**
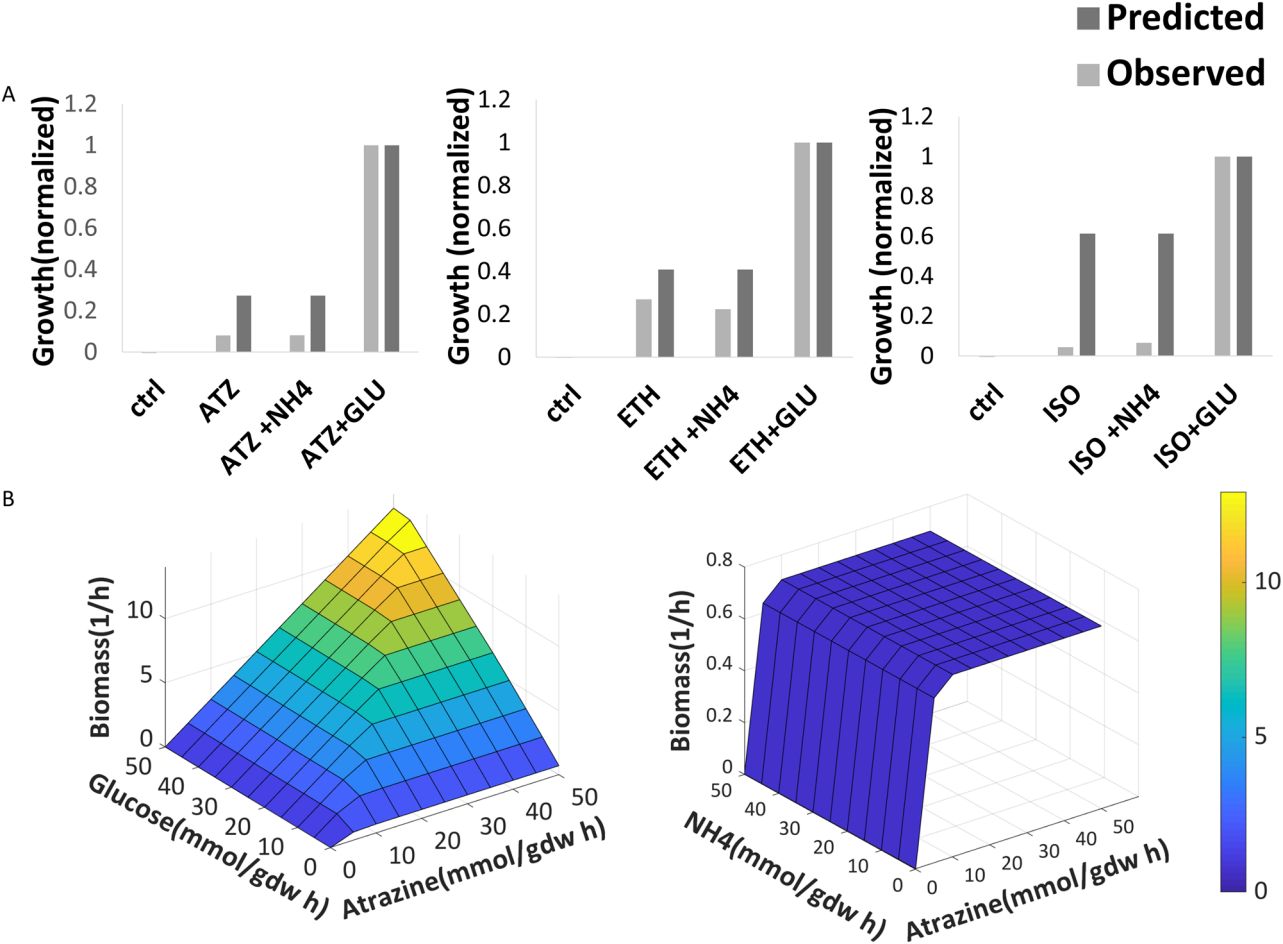
Growth performances of *P. aurescens* TC1 in dependence on the availability of different carbon and nitrogen sources. (**A**) Predicted and observed *P. aurescens* TC1 growth in mineral minimal media supplemented with Atrazine or its degradation intermediates. *ATZ* atrazine, NH4, *GLU* glucose, *ETH* ethylamine, *ISO* isopropylamine, *CTRL* control. Measurements are normalized by dividing in maximal value. Values represent biomass rate (h^−1^, predicted) and OD measurement (observed). (**B**) Surface plot rendering *P. aurescens* TC1 growth (biomass) in dependence on atrazine, glucose and NH4 availability. Color scale is indicative of biomass (Z-axis).

As previously reported, *P. aurescens* TC1 degrades atrazine and metabolizes its intermediates, isopropylamine and ethylamine that can both be used as sole carbon and nitrogen sources. In silico growth simulations in minimal mineral media supplemented by atrazine or one of its degradation intermediates as a sole carbon and nitrogen source confirm that the model captures these reported physiological capacities. Experimental growth measurements verify model simulations and show that atrazine and its degradation products isopropylamine and ethylamine can be used as sole carbon and nitrogen sources for *P. aurescens* TC1 (Fig. 2A). Supplementing the medium with additional carbon (glucose) and nitrogen (ammonium) sources indicate that glucose enhanced growth both in silico and in vitro*,* while the addition of ammonium had not shown an effect on growth (Fig. 2A). This is in accordance with previous work stating that *P. aurescens* TC1 growth is not inhibited by nitrogen sources such as ammonium^26^, unlike the inhibition of atrazine degradation in *P.* ADP in nitrogen rich environments^63^.

Finally, growth simulations under a gradient of nitrogen *vs*. carbon sources further illustrate that atrazine serves as a good nitrogen source and as a poor carbon source (Fig. 2B). The growth promoting potential of combinations of atrazine with ammonium (NH_4_^+^) and glucose as nitrogen and/or carbon sources respectively was tested. The effect of nutrient ratio on growth rate was predicted by optimizing the production of biomass across uptake gradients while constraining the fluxes through exchange reactions for carbon and nitrogen sources. As expected, increasing glucose uptake increased the growth rate (Fig. 2B) and the maximal achievable growth rate was much higher with glucose as a carbon source in comparison to atrazine as a sole carbon source. Increasing ammonium uptake showed no effect, suggesting that atrazine is as equally good nitrogen source as ammonium. This may be endorsed by ammonium as a byproduct of the atrazine degradation process (Fig. 1).

### Media modifications effect on *P. aurescens* TC1 growth

In addition to glucose, several carbon sources including citrate, acetate and sucrose were previously reported to enhance growth of several strains of *Arthrobacter* species in atrazine containing media^64^. To identify additional media modifications that can potentially increase atrazine degradation, we simulated growth in 123 different media combinations, each supplementing the atrazine containing minimal mineral media with a single exchange metabolite (Supplemental Dataset 4). Several sugars, amino acids and dipeptides were predicted to induce enhanced growth. This is in accordance with the previous characterization of *P. aurescens* TC1 as a metabolically versatile species^27,51^.

**Figure 3:**
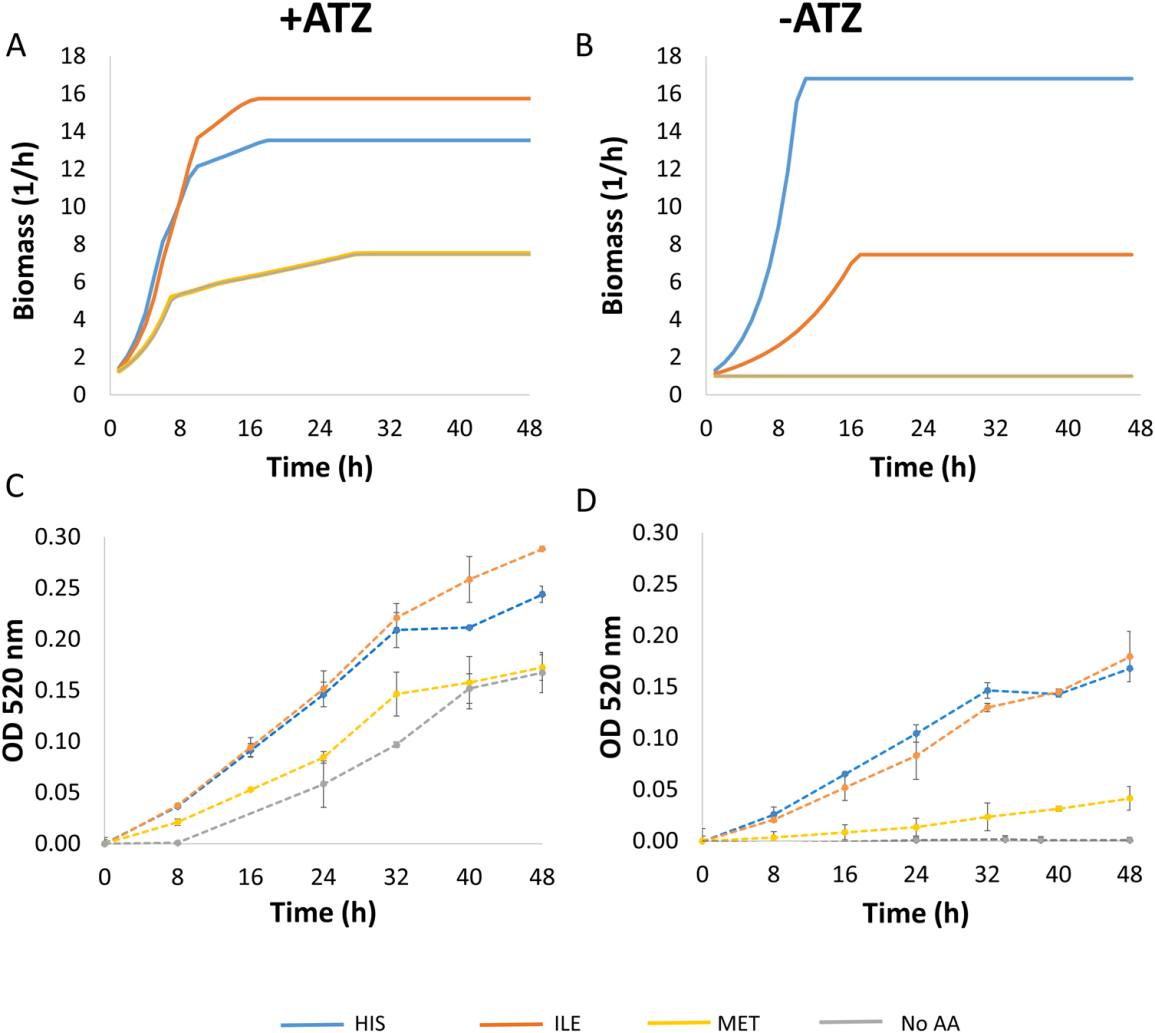
Predicted (**A**,**B**) and observed (**C**,**D**) growth performances of *P. aurescens* TC1 growth in mineral minimal media with glucose complemented by different amino acids, with (**A**,**C**) and without (**B**,**D**) atrazine. *ATZ* atrazine, *HIS* l-histdine, *ILE* l-isoluecine, *MET* l-methionine. Observed values represents mean in triplicates; bars represent SD.

Out of a variety of potential nutritional sources, *Paenarthrobacter* species have been shown to utilize different amino acids in different capacities^65,66^. The flux through the biomass objective function when supplementing media with different amino acids ranges between 1.4 amd 13 h^−1^ (Supplemental Dataset 4). The predicted growth rates when adding l-isoleucine, l-histidine and l-methionine are 11.3, 7.4 and 1.4 h^−1^, respectively, hence representing supplements that are predicted to support growth in high, medium, and low efficiencies. Dynamic simulations of growth while supplementing media with amino acids with or without atrazine was carried for three amino acids whose predicted efficiency in supporting growth ranges from low—l-methionine (biomass flux of 1.4 h^−1^, Supplemental Dataset 4), to moderate—l-histidine (7.4 h^−1^) and high (11.3 h^−1^). Simulation indicated a media dependent effect of these amino acids (Fig. 3). l-Isoleucine has the strongest predicted effect on growth in atrazine containing medium whereas l-histidine has a stronger relative effect in the same medium without atrazine; l-methionine has the weakest effect in all media (Fig. 3). *P. aurescens* TC1 growth in vitro*,* in media corresponding to the media used for the in silico simulations were generally in agreement with model's predictions, supporting the superiority of l-isoleucine over l-histidine in atrazine supplemented media but not in an atrazine deficient medium (Fig. 3). Supplementing media with l-methionine showed a weaker effect on growth in comparison to the other two amino acids tested, with growth in similar level to no amino-acid control, agreeing with its weak effect in simulations. This is also in agreement with a recent work by Deutch^66^ showing that whereas l-isoleucine and l-histidine serve as potential nitrogen and/or carbon source of *P. aurescens* TC1, L-methionine does not serve as a growth supporter. Like atrazine, amino acids can potentially serve as both a carbon and a nitrogen source. Considering C:N ratio as an indicator of growth support potential, l-isoleucine and l-histidine have a ratio of 6:1 and 6:3, respectively, whereas l-methionine, having no growth promoting effect has a ratio of 5:1 (Supplemental Dataset 4). Other amino acids with 5:1 or 5:3 C:N ratio like l-proline and l-serine, respectively, are also predicted to be efficient supporters of growth (biomass flux of ~ 10 h^−1^, similar to l-isoleucine; Supplemental Dataset 4). Thus, the growth effect of atrazine cannot be predicted merely based on C:N ratio.

### Media modifications affect atrazine degradation

**Figure 4:**
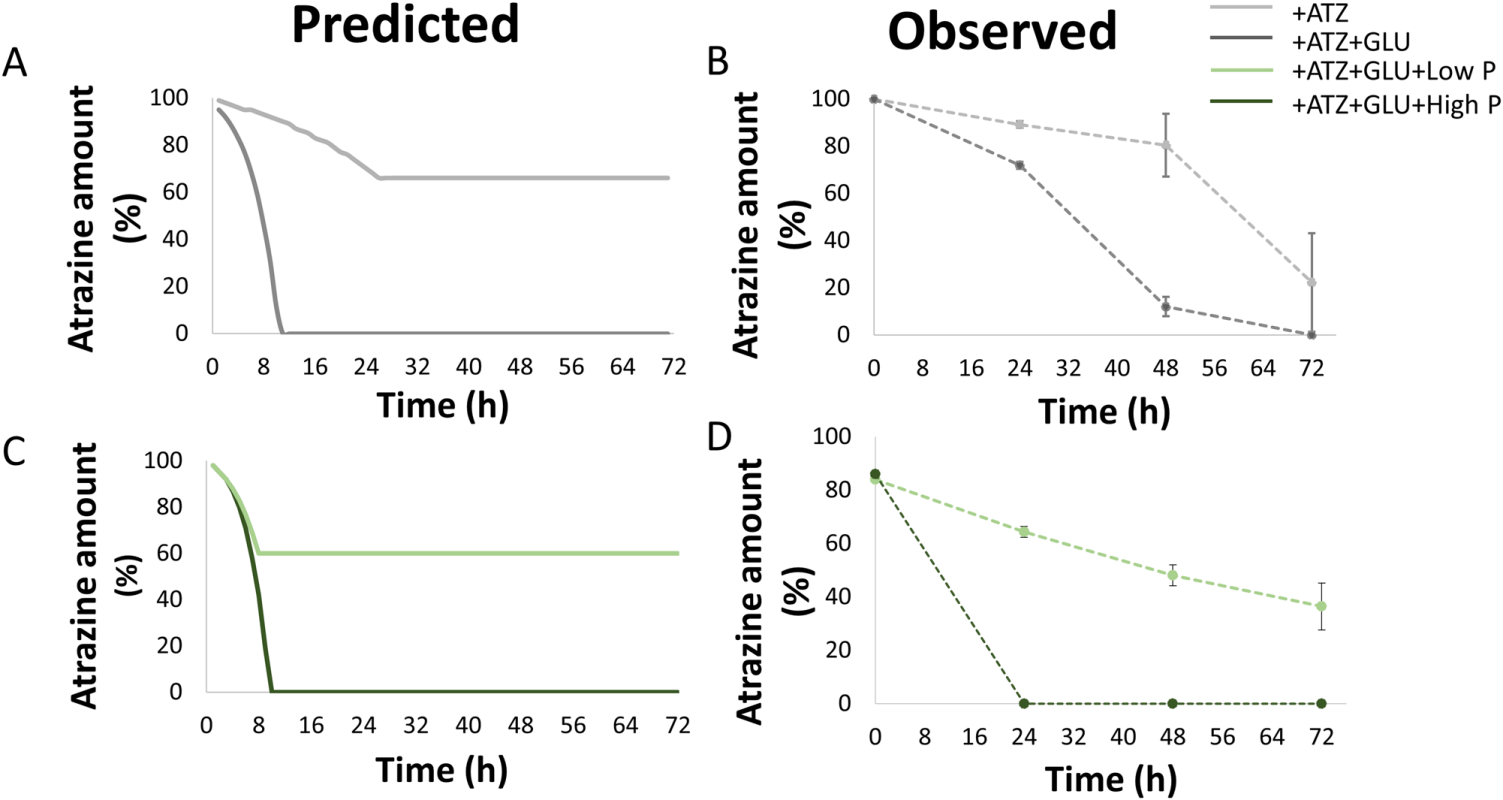
Predicted (**A**,**B**) and observed (**C**,**D**) atrazine degradation in modified media. High and low phosphate concentrations: 50 and 15 mmol/gdw h for predicted values and 0.081 and 0.0081 g/l for observed values, respectively. Initial atrazine amount: 30 mmol (predicted) and 30 mg/l (observed). *ATZ* atrazine, *GLU* glucose. Observed values represents mean in triplicates; bars represent SD.

Following the validation of model predictions for growth capacities, we directly evaluated atrazine degradation in different media conditions. To this end, we applied Flux variability analysis (FVA), allowing the identification of the ranges of flux variability that are possible within a given media^67^. Once the biomass flux was maximized, we estimated the rate of atrazine degradation according to the relevant flux of interest considering several selected medium modifications. The simulation of atrazine degradation over time was in a medium that contains atrazine as a sole carbon and nitrogen source *vs*. modified media sets (see “Methods”). Atrazine degradation was estimated according to the amount of atrazine left in the media following each time cycle. Simulations showed that supplementing the media with glucose not only enhanced growth (Fig. 2) but also induced an enhancement of atrazine consumption. Predictions were confirmed by experimental validation (Fig. 4).

Finally, the addition of phosphate, a main element in fertilizers, to soils has been reported to have a significant effect on the degradation of herbicides. For instance, its addition was shown to effect soil bacteria’s degradation activity including a reported effect on *Arthrobacter* sp. HB-5, a close relative of *P. aurescens* TC1^68^. Phosphate has been previously shown to accelerate atrazine degradation^8^. To test the effect of phosphate, we used the model to simulate growth and atrazine degradation in high or limited phosphate conditions. In accordance with these reports, simulations showed similar trends, which were confirmed by experimental results. These indicate an effect of phosphate concentrations *on P. aurescens* TC1 growth (Supplemental Dataset 5) and atrazine degradation (Fig. 4).

## Discussion

Here, we reconstructed a genome scale metabolic model, *i*RZ1179, of *P. aurescens* TC1, a well-studied atrazine degrader, in order to use the in-silico simulations for predicting optimal media to support its growth and atrazine degradation activity. Model reconstruction was based on automatic pipelines, followed by manual curation processes and adaptation to physiological requirements (feasible growth media). The in-silico simulations were carried on an array of minimal media using atrazine and its interim degradation products as alternative sole carbon and nitrogen sources supplemented by potential enhancers. Simulations predicting and prioritizing specific metabolites as enhancers of growth and degradation were confirmed by in vitro experimental validation. Several limitations of the analysis should be acknowledged. Though the model refinement process included key steps required for construction of high-quality model, further adaptations are required for the current version of *i*RZ1179 to meet the requirements of gold standard models^54^. Mainly, the biomass composition and energy maintenance requirements are approximations that rely on automatic protocols and genomic information^52,53^. Collection of currently lacking experimental data can be incorporated in future versions of the model to define an accurate biomass objective function using, for example, the BOFdat computational platform^69^. Another example for potential improvement is modification of maintenance demands and mass transfer regulation based on a recent detailed account of atrazine degradation in *P. aurescens* at very low concentrations^59^. Second, data used to construct the model are not consistent at their level of confidence and annotations were gathered from numerous databases. A detailed process of manual inspection of each reaction and determination of its substrate/co-factor specificity^54^, was not yet carried. Finally, accuracy of predictions is likely to be significantly improved by the addition of other layers of information including metabolomics, transcriptomics and reaction kinetics^70^. Continuation of the manual curation process, also based on added transcription information, is likely to improve the performances of future versions. Yet, despite limitations, the resulting predictions can successfully capture the relative effect of different media complements on growth and atrazine degradation, as seen in the in vitro experiments performed*,* hence supporting the use of *i*RZ1179 as a predictive tool.

Several key findings emerge from this study. First, simulations successfully capture previous reports on the use of carbon and other sources (glucose and phosphate) as bio-stimulators of atrazine degradation, supported by in vitro validation. Second, the confirmation of *i*RZ1179 as a predictive tool enables fast and low (no)-cost screening and ranking of a wide range of potential nutritional supplements that can serve as stimulators. Here, growth and degradation simulations were carried with more than 100 compounds consumed by *P. aurescens* TC1. In vitro validation confirmed the predicted classification of different amino acids as efficient, moderate or poor stimulators of degradation activity. Simulation outcomes cannot be pre-determined based solely on the C:N ratio of media supplements but rather consider the complementary stoichiometric effect of multiple chemical reaction cascades, or pathways, that construct the genome scale metabolic network. Thus, these describe the complexity of cellular processes and balance between different metabolic pathways contributing to growth. Simulations per se provide multiple optimal solutions for the internal fluxes involved in primary metabolism and hence are not sufficient for deciphering the mechanism of how environmental (media dependent) conditions affect atrazine degradation. However, integration of simulation data with `omics data (e.g., metabolomics, proteomics, and transcriptomics) can reduce the solution space and shed light on the fine-tuning of cellular activity considering the need to balance between myriads of constrains. Beyond the current study, the model can serve the contextualization of results from a growing number of high throughput experiments describing the relative abundance of discrete elements (e.g., genes, enzymes) into a higher level understanding of cellular activity. For example, future integration of proteomic data as produced in a recent study exploring adaptation of *P. aurescens* TC1 to growth in atrazine-fed bioreactors^59^, can serve for both model refinement and as a predictive tool.

Finally, model predictions can go beyond supplementing the medium with a single compound. Here, we demonstrated that the efficiency of the combination of different amino acids as growth enhancers depends on other medium components (here –atrazine). Hence, the analysis demonstrates that the exhaustive power of the genome scale metabolic reconstruction allows capturing complexities that are beyond common biochemical expertise and knowledge and further support the importance of computational platforms for the educated design of complex media.

The in vitro validation provides a proof of concept for the use of *i*RZ1179 as a predictive tool and can be considered as a first step towards designing bio-stimulation strategies that can be further tested in vitro and in the field. Soil communities are complex in terms of composition and microbial consortia dynamics, hence imposing greater complexity. Despite the complexity of soil communities, supplementing soil with carbon sources is long known to induce a change in microbial community structure and function. In a recent study we demonstrated the usefulness of modeling approaches for deciphering metabolic complexities within indigenous soil community treated with atrazine^49^. The integration of species-specific models together with community-based modelling approaches is likely to contribute to our understanding of the biodegradation processes of atrazine in natural environments and promote the development of bioaugmentation and biostimulation strategies. More generally, soil contaminations caused by pesticides and herbicides are considered among the top ten environmental hazards, with no current solutions that support green and cost-effective soil detoxification processes^71^. Exploiting the exhaustive power of the computational modeling platforms allows for a relatively fast search for bioremediation solutions that optimize the degradation activity of the existing natural communities, by allowing screening of hundreds of thousands of possible solutions and scoring them. Simulations can be designed to screen for metabolites, microbial species and combinations of both. Finally, in order to develop realistic solutions, a potential bio-stimulator should not only be efficient but also cost effective, ruling out expensive degradation enhancing compounds such as dipeptides. The ultimate proof of concept for the advent of computational approaches should be the design of cost-effective bioremediation strategies that outperform solutions that can be found by standard ‘trial and error’ practices.

## Methods

### Reconstruction and curation of the *P. aurescens* TC1 metabolic model

An initial draft model of *P. aurescens* TC1 was reconstructed using automatic pipeline, as described in Henry et al.^52^. Briefly, the complete genome sequence of *P. aurescens* TC1 was retrieved from NCBI (GenBank accession IDs. NC_008711 including NC_008711.1 NC_008712.1, and NC_008713.1—chromosome and two plasmids, respectively), uploaded into RAST^55^, spliced into genes and annotated. RAST annotations were put through the Model SEED pipeline^52^ and an initial metabolic network reconstruction was obtained. Model refinement included addition of reactions identified in the specific strain in literature and additional databases, validation of reactions’ elemental balance and directionality, removal of futile loops and gap filling. Simulation conditions were modified to reflect experimentally validated minimal media alternatives that support growth of *P. aurescens* TC1^24^. All simulations were carried out in a Minimal Mineral Media (MMM: K^+^, Mn^2+^, CO_2_, Zn^2+^, SO_4_^−^, Cu^2+^, Ca^2+^, HPO_4_^2−^, Mg^2+^_,_ Fe^2+^, Cl^−^), supplemented by alternative carbon and nitrogen sources including atrazine, ethylamine, isopropylamine, ammonium, glucose and selected amino acids. The model is available in SBML format and as excel sheet (Supplemental Datasets 1 and 2, respectively). To improve model comparability, cross-references to the BiGG databases were added containing intuitive namespaces for reactions and metabolites^72^ (Supplemental Dataset 1). The minimal media used for simulations are available in Supplemental Dataset 3.

### Flux balance analyses simulations and dynamic, time dependent simulations

Flux distributions in the metabolic model were determined using Flux Balance Analysis (FBA)^54,73,74^. Briefly, a metabolic model includes a network that is described as a stoichiometric matrix **S**_*m*×*n*_, where *m* represents the number of metabolites and *n* the number of reactions in the model. In the assumed pseudo-steady-state, internal metabolites concentrations and fluxes are assumed to be constant. The model can be represented as: **S** × v = 0, where vector v signifies the fluxes through the internal reactions. As the number of reactions (*n*) typically exceeds the number of metabolites (*m*), the system is under-determined. In order to get a feasible solution space, linear programming (LP), subject to mass balance preservation and flux constraints, was used by introducing an optimization problem. Here, model growth was maximized by maximizing its biomass reaction, which was used as objective function. Specific fluxes for metabolites consumption and secretion were determined following fixation of the respective biomass reaction to its maximal value and then using Flux Variability Analysis (FVA)^67^, a linear programming method to find the minimal amount of metabolites needed to produce a preset amount of biomass. Dynamic modeling was used for the prediction of the profile of consuming metabolites typical to the biomass increase of *P. aurescens* TC1 and atrazine degradation across time. To this end, we simulated the behavior of our metabolic model across time. This was done as described in^49^ and illustrated in Supplemental Dataset 5, based on concepts defined in^75^. Briefly, the model works under the following assumptions: (1) a finite start amount of media components is available; (2) a maximal amount of uptake a single cell can acquire from the media in a given time point is defined (the lower bound of the exchange reaction value); (3) new substrate concentrations in time t, are determined by the predicted substrate concentration for the previous step augmented with any additional substrates provided or consumed in the current iteration. The maximum uptake was set to a ratio of up to 1 unit of each metabolite available in the media; (4) after each time tick, the biomass amount was updated according to the flux amount of the biomass reaction in the model at this time tick. As biomass production rate serves as a proxy for the size of the population in the simulated environment and substrate uptake/secretion is mainly affected by to population size, the model was used to evaluate the actual substrate uptake/secretion and growth rate given the supplied media across time. Simulations were carried until reaching a state where time cycles did not lead to an increase in biomass. Initial concentration values for all metabolites were set to a fixed amount of 50 units (represented as the initial lower bounds, LB, of the exchange reactions). Simulation parameters and defaults are available in Supplemental Dataset 3. Starting with one bacterial cell, the flux balance model was used to predict the uptake of phosphate, sulfate, carbon and nitrogen sources (i.e. atrazine and glucose) by *P. aurescens* TC1 across time.

All model simulations were done on an Intel i7 quad-core server with 32 GB of memory, running Linux. The development programming language of our simulators was JAVA, and our linear programming software was IBM CPLEX. Surface plots were created using the MATLAB 2019a (MathWorks, Inc., Natick, Massachusetts, United States). Pathway maps were created using Escher maps^76^.

### In vitro bacterial growth conditions and chemical analysis

*Paenarthrobacter aurescens* TC1 (ATCCBAA-1386) was grown on R medium (based on ATCC 2,662 R) plates with glucose (0.2%) and atrazine (30 mg/l, Agan Adama-Ashdod, Israel) as sole nitrogen. Stock cultures were maintained in 15% glycerol at − 80 °C. Cultures were resuscitated on R medium agar plates (1.5% Difco bacto) and were used as starter colonies for liquid medium growth experiments. The growth of *P. aurescens* TC1 was tested in R media supplemented with different combinations of carbon and nitrogen sources, including Glucose (0.2%), Atrazine (30 mg/l, Agan Adama-Ashdod, Israel), ammonium chloride (37 mg/l), Ethylamine and Isopropylamine (31.1 and 40.8 mg/l, Sigma-Aldrich-Rehovot, Israel) and the amino acids Histidine, Isoleucine and Methionine (36, 92 and 103 mg/l respectively). Concentrations of amino acids, ethylamine and Isopropylamine were calculated to provide the same nitrogen content as the atrazine. In addition to the carbon and nitrogen sources, growth in media supplemented by atrazine and atrazine together with glucose was tested under two phosphate concentrations (K_2_HPO_4_ in 0.45 and 0.045 g/l). Each substrate was added to sterile R medium and bacteria were grown in triplicate 250 ml flasks incubated at 30 °C with shaking in the dark. One flask contained only R medium and was used as control. A 200 µl sample was taken from each flask every 8 h for measurement of OD in microplate reader (520 nm, Infinite 200 PRO Tecan, Männedorf Switzerland) and 1 ml was taken to study atrazine degradation rate using HPLC. The HPLC analysis was performed with Agilent HPLC (Waldbronn Germany) equipped with a DAD detector. Samples were separated on Kinetex C18 column (Phenomenex Torrance, CA) and the mobile phase consisted of 70% methanol flowing at 1 ml/min. Detection and quantitation of atrazine was done at 240 nm with detection limit of 0.1 mg/l. Total duration of each growth experiment was between 152 and 168 h.

## Supplementary information

Supplementary Dataset 1.

Supplementary Dataset 2.

Supplementary Dataset 3.

Supplementary Dataset 4.

Supplementary Dataset 5.
