# Supplementary material for: Genome-scale reconstruction of Paenarthrobacter aurescens TC1 metabolic model towards the study of atrazine bioremediation
Source: Sci Rep. 2020 Aug 3;10:13019. doi: 10.1038/s41598-020-69509-7 (PMC7398907; doi:10.1038/s41598-020-69509-7)
Supplement: Supplementary file 5 — Supplementary Dataset 5. [file 41598_2020_69509_MOESM5_ESM.docx]

# Supplemental data 5 - Genome-Scale reconstruction of *Paenarthrobacter aurescens* TC1 metabolic model towards the study of atrazine bioremediation

Shany Ofaim^1,2^* ^ᵻ^, Raphy Zarecki^1^*, Seema Porob^3^, Daniella Gat^3^, Tamar Lahav^1^, Yechezkel Kashi^2^, Radi Aly^1^, Hanan Eizenberg^1^, Zeev Ronen^3 ϯ^ & Shiri Freilich^1ϯ^

^1^Newe Ya'ar Research Center, Agricultural Research Organization, Ramat Yishay, Israel, ^2^Faculty of Biotechnology and Food Engineering, Technion-Israel Institute of Technology, Haifa, Israel, ^3^Department of Environmental Hydrology & Microbiology, Zuckerberg Institute for Water Research, Jacob Blaustein Institutes for Desert Research, Ben-Gurion University of the Negev, Midreshet Ben-Gurion, 8499000, Israel

*equal contribution





Figure S1 – *P.aurescens* TC1 growth in high and limited (low) phosphate conditions. High and low phosphate concentrations: 50 and 15 mmol/gdw h for predicted and 0.081 and 0.0081 g/l for observed, respectively. ATZ- atrazine, GLU- glucose. Observed values represents mean in triplicates; bars represent SD.


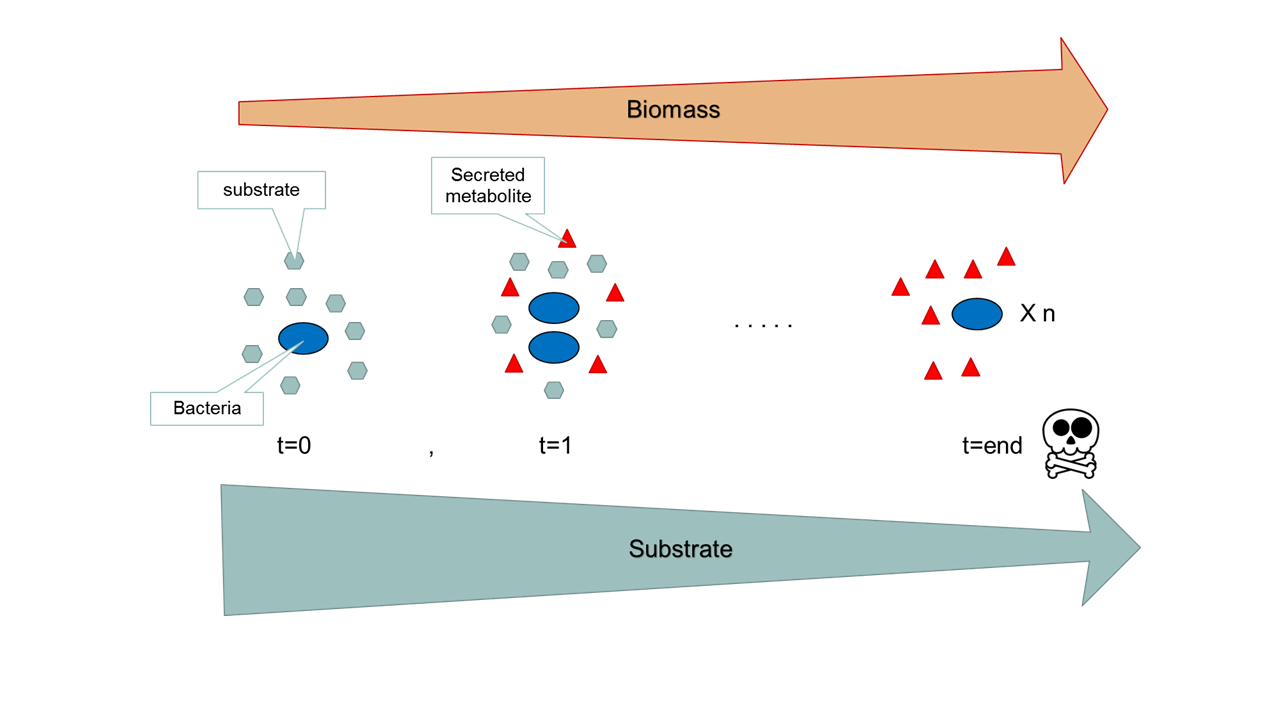


Figure S2 - A schematic diagram describing a time dependent simulation of nutrient dependent bacterial growth.
